# Letter from Dr. W. W. H. Thackston to the Southern Dental Association

**Published:** 1869-10

**Authors:** W. W. H. Thackston


### CORRESPONDENCE.

### ARTICLE V.

### *Letter from Dr. W. W. H. Thackston to the Southern Dental Association.*

Farmville, July 21st, 1869.

W. H. Morgan, M. D., D. D. S.

Dear Sir :—Please accept my thanks with my acknowledgement of the " Circular " inviting me to participate with yourself and others in the effort to organize a " Southern Dental Association," &c.

I extremely regret to say that in consequence of severe indisposition in my family I shall not be able to be with you in Atlanta, but allow me to assure you that I am and will be with you and your confrerés in full and cordial sympathy —in earnest wishes for the success of your laudable enterprise, and in sanguine convictions as to the great good to result from the movement you are about to inaugurate.

I have long cherished the design of attempting such an organization of Southern Dentists as you now contemplate, but, from various causes, have deferred moving in the matter. I now rejoice that the initiative has been taken, and as the "Southerns" have at last "put their hands to the plough " I trust there will be no looking back.

There is no egotism in the assertion that we have talent, skill, education and energy in the South to build up and sustain an Association that will not only honor ourselves, but will shed lustre upon our chosen calling and keep Southern Dentistry fully abreast with all the sciences in the onward and upward march to light and perfection.

Please in my behalf utter greetings heartfelt and strong to my brethern of the craft. All hail ! My Brethern ! God speed your glorious work ! Allow me to stimulate your zeal, excite your enthusiasm and to nerve your energies for the to grand and noble task you have assumed. We have the material for a splendid Edifice ; we have skilled workmen with cunning hands ; we have what is better than the cedar of Lebanon, the purple of Tyre or the gold of Ophir ; we have a profession fully up in its requirements and its demands, to the most exalted abilities, and we have a land and a country that will sustain and requite the proudest achievements of genius.

What more inviting field ? What more inspirating prospects could we desire ? What brighter promises ever lit up the pathway of Science, or inspired the hearts of its votaries ? And what stronger motives ever incited human action than now appeals to our Southern "Esprit des Corps" ?

I am now a little past the meridian of life, nearly 28 years ago I received the 3rd Diploma, that was issued by the *first* and then the only Dental College in the world. Dr. Arthur, of Baltimore, and Dr. Mackall are the only two graduates of a Dental College older than myself, and they preceded me only one year, they graduated at the *first commencement* of the Baltimore College of Dental Surgery, and I at the second. I am the only surviver of my class, and of the Faculty whose instructions I received, Dr. Thos. E. Bond is now the sole surviver. I have lived to see nearly all my early associates, students and teachers, pass away, but I hope I may yet be spared to see my loved and native South at least professionally unshackled and disenthralled ; to see her with her own well sustained Dental Institutions, her own Laboratories, and her own Literature.

I regard the movement you are now inaugurating as one of the most vital importance to Dental Surgery in the South ; as one fraught with the gravest consequences, as one pregnant with good or evil for our loved calling and our no less loved country. There must be no failure. We must *achieve a success*, and to that end I am with you *heart and soul*, according to the measure of my humble capacity. I am ready to serve you, and to co-operate with you in any way that may conduce to, or promote the objects and purposes of the Association.

To prevent any possible misapprehension, I beg leave just here to say, that *I am not a candidate for any of the offices or honors of the Association*, the post of "high private in the ranks" is the only position I aspire to, for in that capacity I am ready to serve the Association to the best of my ability.

With sincere wishes for your success, and highest regards for you and your associates personally, I have the honor to be,

Your Ob'd't. Serv't,
W. W. H. Thackston.

---

